# Supplementary material for: Thermal annihilation of photo-induced radicals following dynamic nuclear polarization to produce transportable frozen hyperpolarized 13C-substrates
Source: Nat Commun. 2017 Jun 1;8:15757. doi: 10.1038/ncomms15757 (PMC5461505; doi:10.1038/ncomms15757)
Supplement: Supplementary Information — Supplementary figures, supplementary note, supplementary methods and supplementary references. [file ncomms15757-s1.pdf]

SUPPLEMENTARY METHODS

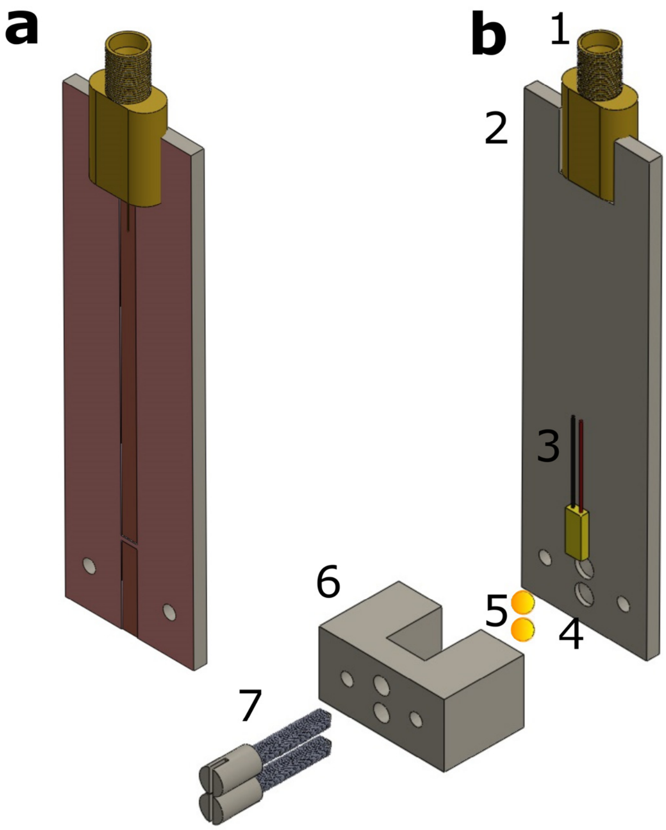

Supplementary Figure 1. ESR setup for measuring the radical quenching temperature. a) Front view of the ESR probe. The 9.5 GHz half-wavelength coplanar waveguide resonator (dark reddish brown) is printed on a grounded 37-µm thick copper layer (light reddish brown). **b)** Back view of the ESR probe. The main elements are: SMA connector (1), PCB board (2), PT1000 resistance (3), two 2.5-mm diameter hemispherical cavities designed to host two spherical samples (4), frozen beads (5), polyoxymethylene bridge (6) and screws (7) to maintain the beads in place; the screws used to connect the bridge to the PCB board are not shown.

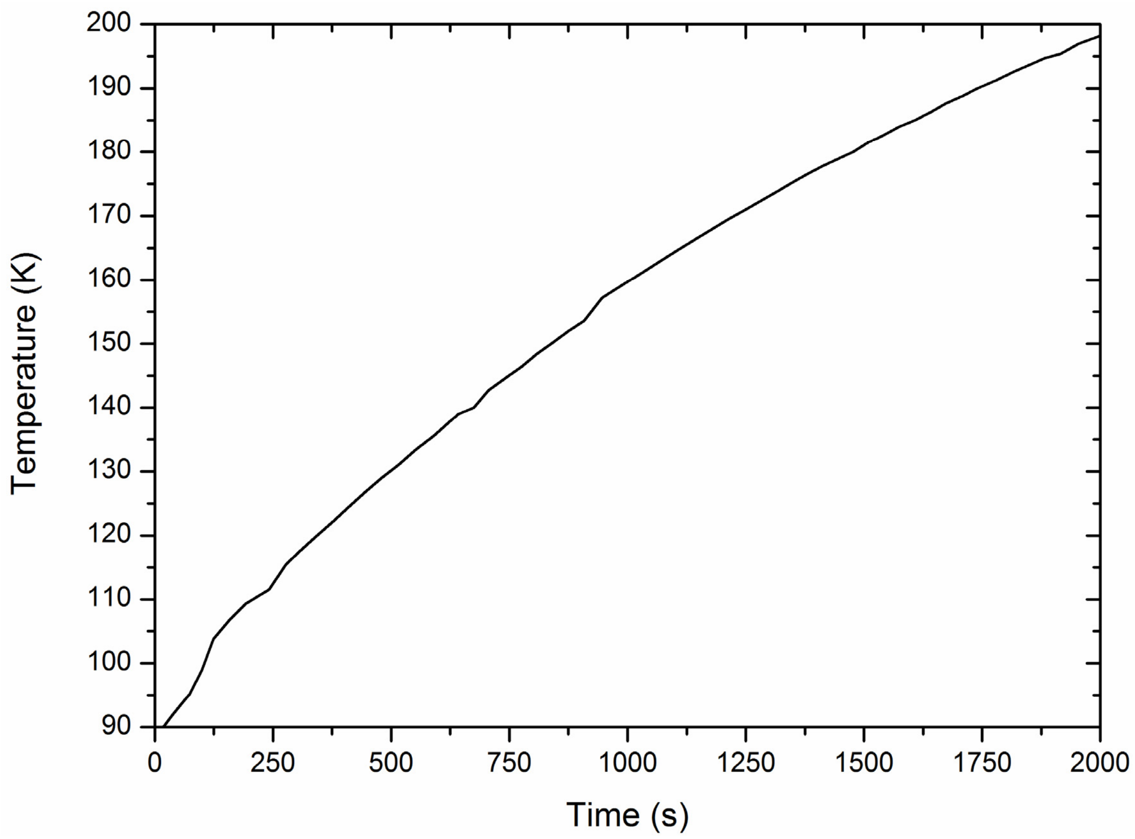

Supplementary Figure 2. UV-induced radical quenching temperature. Temporal evolution of the ESR probe temperature within the ESR cryostat during unregulated warm-up.

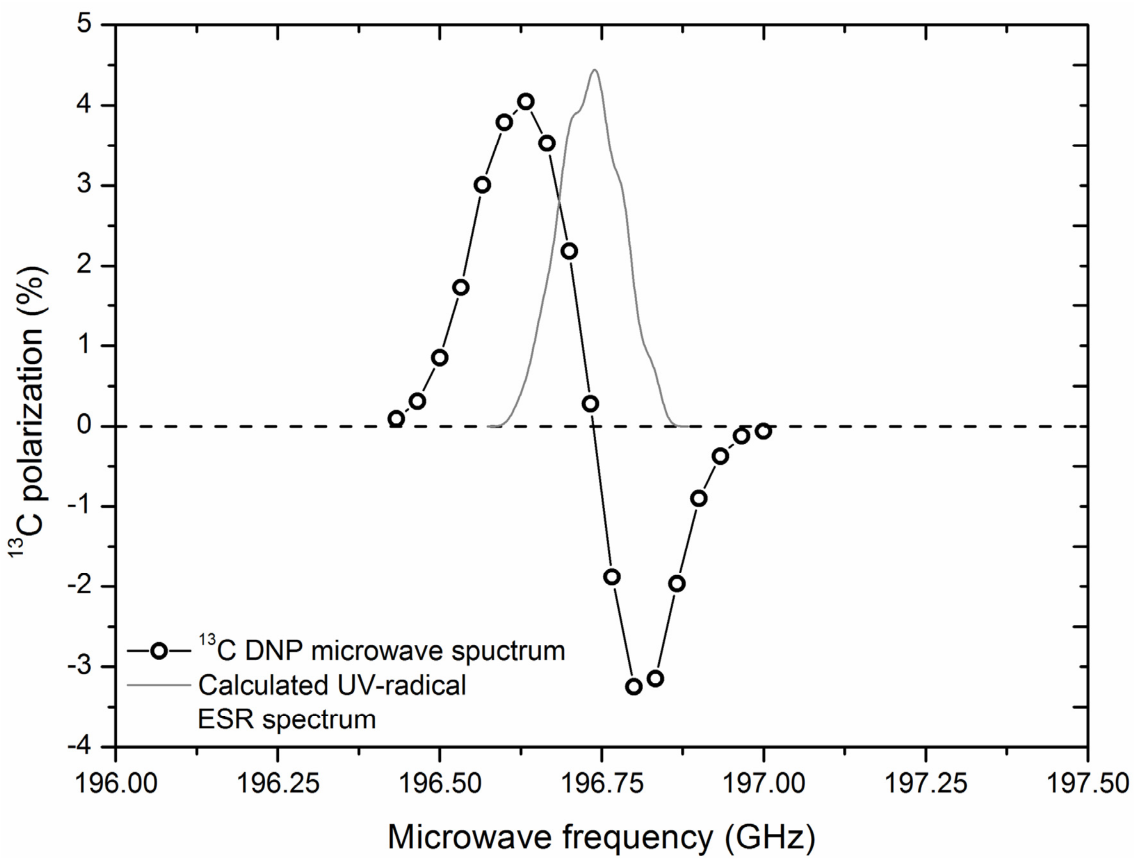

Supplementary Figure 3. DNP microwave spectrum. ^{13}C DNP microwave spectrum (open circles connected by black segments) measured at 7 T and 4.2 K in UV-irradiated [1-^{13}C]PA:H_2O 1:1 (v/v). The ^{13}C polarization was determined for each microwave frequency after 60 min of irradiation (microwave output power set to 55 mW) using the method described in the main text (see Methods). The grey curve represents the ESR spectrum of the photo-induced radical calculated at 7 T with the PEPPER routine of the MATLAB®-based software EasySpin[1] using the g-tensor ([2.0041 2.0037 2.0042]), ^{1}H hyperfine coupling (48 MHz isotropic to each of the 3 methyl group protons), ^{13}C hyperfine coupling (30 MHz isotropic), and Gaussian line broadening (20 MHz) values obtained from X-band ESR measurements (see Supporting Information in ref. 2, DOI: 10.1021/acs.jpcc.5b07315).

Solid-state ^{13}C NMR coil calibration and rf correction

UV-irradiated [1-^{13}C]PA:H_2O 1:1 (v/v) beads were partially polarized via DNP at 7 T and 4.2 K with microwave irradiation at 196.633 GHz (55 mW). Once the microwave irradiation was stopped, single-scan ^{13}C NMR acquisitions were recorded during the application of a series of single-pulse rf excitations (5 μs at 75.100 MHz; 6.26 W) equally spaced in time (1 s) (Supplementary Fig. 4). The decay curve was fitted with the following expression: $S(n) = S_0\left[\cos\left(\frac{2\pi}{360°}\theta\right)\right]^{n-1}$ where $S(n)$, S_0, and θ respectively represent the NMR signal integral recorded during acquisition number n, the NMR signal integral recorded during the first acquisition, and the pulse flip angle, the latter being the only free parameter. The fitted curve provided $\theta = 6 \pm 0.5°$ and this value was used to correct both the build-up and the longitudinal relaxation time constants for the effect of the rf pulses using $\frac{1}{\tau} = \frac{1}{\tau'} + \frac{\ln(\cos\theta)}{T_R}$, where τ' is the apparent time constant, τ the corrected time constant, and T_R is the time interval between two consecutive pulses.

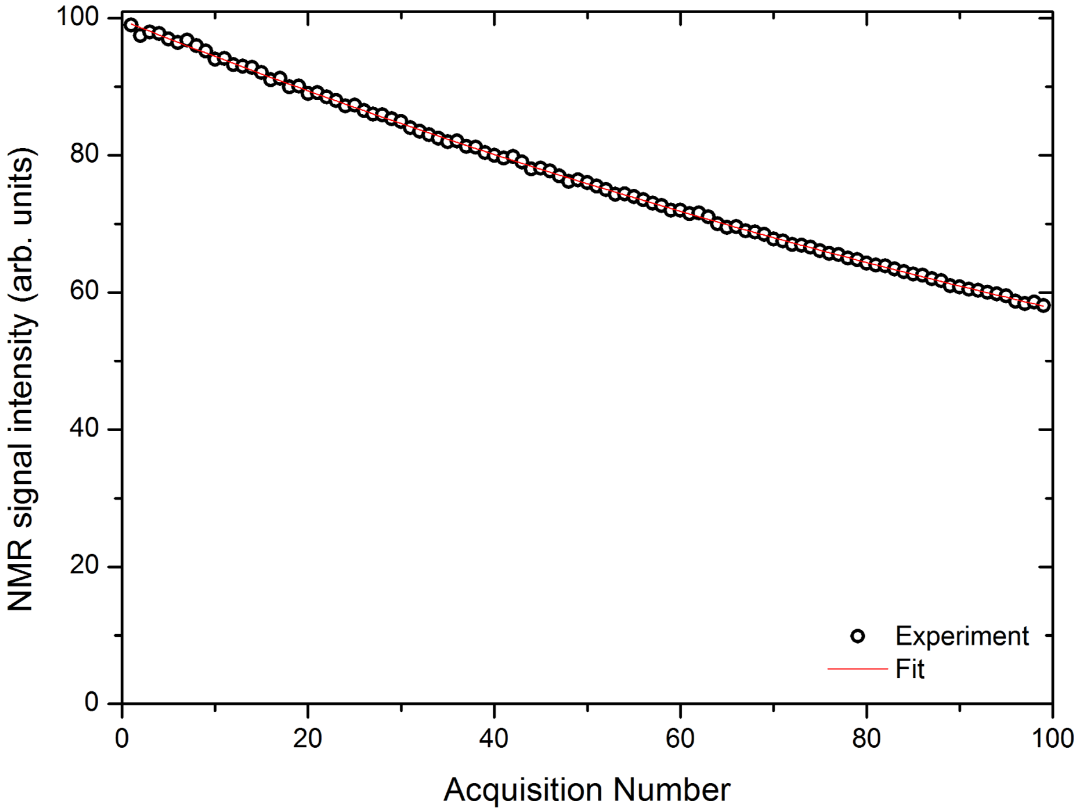

Supplementary Figure 4. ^{13}C NMR signal integral as a function of the acquisition number. The consecutive single-scan acquisitions spaced by 1 s. rf excitation was done with a single 5 μs and 6.3 W square pulse, corresponding to a flip angle of 6.0±0.5°). The measurements were performed at 4.2 K and 7 T after having partially polarized the sample for 10 min with 55 mW microwave power (as measured at the source output) at 196.633 GHz (optimal frequency according to Supplementary Fig. 3).

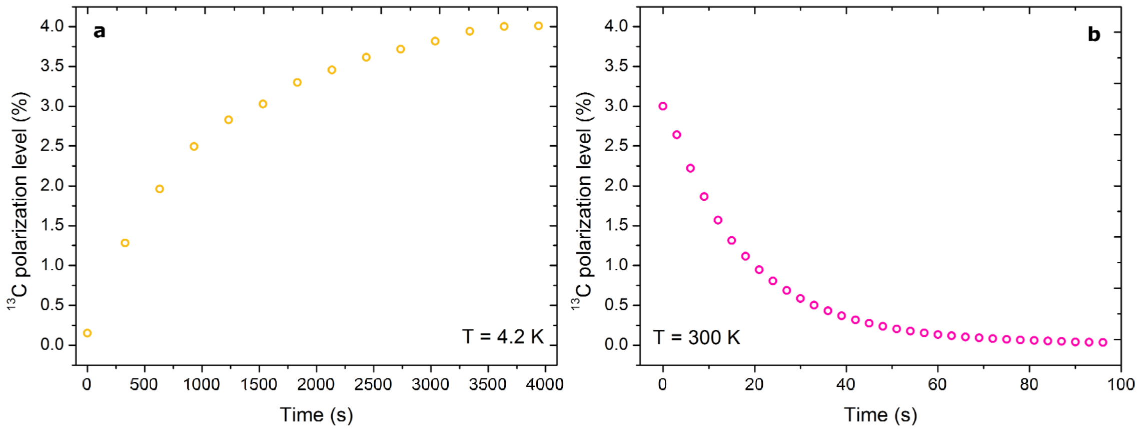

Supplementary Figure 5. ^{13}C polarization evolution during DNP at 7 T/4.2 K and after *ex situ* dissolution. a) Solid-state ^{13}C polarization time evolution under microwave irradiation (196.633 GHz) at 4.2 K and 7 T. **b)** Liquid-state ^{13}C polarization time evolution after thermalization and *ex situ* dissolution measured at room temperature inside a 9.4 T MRI scanner.

SUPPLEMENTARY NOTE 1

Theoretical energy required for thermalization process

The 4 µL spherical beads (~2 mm diameter) are formed of pyruvic acid (PA) and H_2O 1:1 (v/v), i.e., 2 µL of PA and 2 µL of H_2O. The density of PA is 1.281 g/ml, so that 2 µL is equivalent to 1.281·2 = 2.562 mg. Since 1 mol of PA is equivalent to 89.05 g, 2 µL PA corresponds to 29 µmol. The density of H_2O is 1 g/mL, so that 2µL is equivalent to 2 mg. Since 1 mol H_2O is equivalent to 18 g, 2 µL H_2O corresponds to 111 µmol.

The heat capacity of pyruvic acid is C_P = 114.6 J/(mol·K)[3]. To bring 29 µmol of PA from 1 K to 190 K, an energy of $\Delta E = 114.6 \cdot (190-1) \cdot 29 \cdot 10^{-6} \cong 0.63\,\text{J}$ is necessary. The heat capacity of water can be averaged to about C_P = 20 J/(mol·K) below 190 K. To

bring 111 μmol of H_2O from 4.2 K to 190 K, an energy of $\Delta E = 20 \cdot (190 - 1) \cdot 111 \cdot 10^{-6} = 0.42$ J is necessary.

Therefore, a total of about **1 J** is necessary to bring the bead temperature from 1 K to 190 K.

To melt 2 μL of PA, it is necessary to bring the PA temperature to 284 K and an additional energy of 10.81 kJ/mol (enthalpy of fusion): $\Delta E = (114.6 \cdot (284 - 1) + 10810) \cdot 29 \cdot 10^{-6} \cong 1.25$ J. To melt 2 μL of H_2O, it is necessary to bring the water temperature to 273.2 K and an additional energy of 6.01 kJ/mol (enthalpy of fusion): $\Delta E = (20 \cdot (273.2 - 1) + 6010) \cdot 111 \cdot 10^{-6} \cong 1.27$ J.

Therefore, a minimum of **2.5 J** is necessary to melt a bead.

References

1 Stoll, S. & Schweiger, A. EasySpin, A Comprehensive Software Package for Spectral Simulation and Analysis in EPR. *J. Magn. Reson.* **10**, 42−55 (2006).

2 Capozzi, A. *et al.* Photoinduced Nonpersistent Radicals as Polarizing Agents for X-Nuclei Dissolution Dynamic Nuclear Polarization. *J. Phys. Chem. C* **119**, 22632-22639 (2015).

3 Dalla-Betta P. & Schulte M., Calculation of the Aqueous Thermodynamic Properties of Citric Acid Cycle Intermediates and Precursors and the Estimation of High Temperature and Pressure Equation of State Parameters. *Int. J. Mol. Sci.* **10**, 2809-2837 (2009).
